# Biotechnological Approaches on Two High CBD and CBG *Cannabis sativa* L. (Cannabaceae) Varieties: In Vitro Regeneration and Phytochemical Consistency Evaluation of Micropropagated Plants Using Quantitative ^1^H-NMR

**DOI:** 10.3390/molecules25245928

**Published:** 2020-12-15

**Authors:** Kostas Ioannidis, Evangelos Dadiotis, Vangelis Mitsis, Eleni Melliou, Prokopios Magiatis

**Affiliations:** 1 Laboratory of Sylviculture, Forest Genetics and Biotechnology, Institute of Mediterranean and Forest Ecosystems, Hellenic Agricultural Organization “Demeter”, Ilissia, 11528 Athens, Greece; 2 Department of Pharmacognosy and Natural Products Chemistry, Faculty of Pharmacy, National and Kapodistrian University of Athens, Panepistimiopolis Zografou, 15771 Athens, Greece; vaggdad@gmail.com (E.D.); emelliou@pharm.uoa.gr (E.M.); magiatis@pharm.uoa.gr (P.M.); 3 Ekati Alchemy Lab SL, 08180 Barcelona, Spain; ekatimed@gmail.com

**Keywords:** *Cannabis sativa*, Cannabaceae, in vitro micropropagation, cannabinoids, cannabidiol, cannabigerol, chemical fidelity, quantitative NMR

## Abstract

High cannabidiol (CBD) and cannabigerol (CBG) varieties of *Cannabis sativa* L., a species with medicinal properties, were regenerated in vitro. Explants of nodal segments including healthy axillary bud, after sterilization, were placed in Murashige-Skoog (MS) culture medium. The shoots formed after 30 days were subcultured in full- or half-strength MS medium supplemented with several concentrations of 6-benzyl-amino-purine (BA) or thidiazuron (TDZ). The highest average number and length of shoots was achieved when both full and half-strength MS media were supplemented with 4.0 μM BA. The presence of 4.0 μM TDZ showed also comparable results. BA and TDZ at concentrations of 4.0, 8.0 μM and 2.0, 4.0 μM respectively, displayed the maximum shooting frequency. The new shoots were transferred on the same media and were either self-rooted or after being enhanced with different concentrations of indole-3-butyric acid (IBA) or α-naphthalene acetic acid (NAA). Presence of 2.0 or 4.0 μM IBA or 4.0 μM NAA resulted to the optimum rooting rates. The maximum average number and length of roots per shoot was observed when the culture media was supplemented with 4.0 μM IBA or NAA. Approximately 92% of the plantlets were successfully established and acclimatized in field. The consistency of the chemical profile of the acclimatized in vitro propagated clones was assessed using quantitative ^1^H-NMR high throughput screening. In each variety, analysis of the micropropagated plant in comparison with the mother plant showed no statistically significant differences (*p* ≤ 0.05) in CBD+ cannabidiolic acid (CBDA) and CBG+ cannabigerolic acid (CBGA) content respectively, thus indicating stability of their chemical profile.

## 1. Introduction

The Greek physician and pharmacologist Pedanius Dioscorides had already observed the effectiveness of the infusion of *Cannabis*’ green parts for otalgia treatment (ear-ache) in his timelessness medical work, “De materia medica” (70–77 A.D.). His ancient script is a guide to ancient medicines which describes the medical uses of plants (Dioscorides *Materia* medica 3.149.1) [1]. Both Ancient Greeks and Romans noted the medicinal properties of *Cannabis* [2], in contrast to Ancient Egyptians [3], although the first appearance of *Cannabis* is believed to be in central Asia about 5000 B.C. [4] or even earlier [3]. According to Schultes et al. [5] it is one of the oldest domestic plants in the history of mankind and has been cultivated for at least 10,000 years.

All these centuries, *Cannabis* was mainly used for fiber (mats, shoes, cloth, and ropes) and oil production. As mentioned by Salami et al. [6], over 25,000 different products have been derived and used for various purposes from *Cannabis* plant. However, the species was less used for its pharmaceutical action.

A number of compounds with medicinal properties, such as terpenoids, flavonoids and phytosterols [7], alkaloids and glycoproteins [8], are present in cannabis. Also, there is a class of terpenophenolic compounds which is uniquely produced by *Cannabis* plants and that is the cannabinoids [9]. The phytocannabinoids are mainly synthesized in secretory cells inside glandular trichomes which are present on the female flowers and fruits of cannabis plant [10,11].

Over 480 compounds have been reported from cannabis plants [12], although most of them have neither been isolated nor characterized [13]. About 150 compounds are considered, in the basis of the chemical structure, as phytocannabinoids [14,15,16]. The most studied phytocannabinoids concerning their therapeutic uses are the intoxicating Δ^9^-tetrahydrocannabinol (Δ^9^-THC), a promising medicinal compound for treating various diseases [17] with well-known medicinal effects [18], and cannabidiol (CBD) which several proven pharmacological properties [19,20].

In addition to former substances, Δ^9^-THC and CBD, several cannabinoids, for instance cannabigerol (CBG) [21,22,23,24], are being investigated for their potential medicinal effects. Such substances are not abundant in cannabis plants. However, cannabis varieties or strains that produce high amounts of these minor cannabinoids have been detected. CBG-enriched [25] and CBD-enriched [26] varieties have already been described. In recent years, agricultural genetists and breeders have selected several cannabis varieties, that predominantly produce high amounts of CBD [27], cannabidivarin (CBDV) [28] and CBG [29]. This genetic selection would enable the production of varieties rich in specific phytocannabinoids [13]. Moreover, it is very important, through the breeding steps, to carry out chemical screening of cannabis varieties and investigate their phytochemical profile stability.

Currently, cultivation and breeding drug-type (THC-rich) chemical phenotypes of *Cannabis* is prohibited in most countries, with the exception of research purposes and pharmaceutical uses [12] due to the medicinal effects of Δ^9^-tetrahydrocannabinol’s [18]. Because of this prohibition, breeders have turned to the discovery and identification, or even breeding, of fiber-type varieties rich in non-psychotropic cannabinoids with medicinal activity. Moreover, they propagate such CBD- or CBG-enriched varieties through vegetative propagation in order to increase the minor phytocannabinoids production.

Having as target the large-scale propagation of the two selected and screened varieties with desirable characteristics, the first one rich in CBD and the second in CBG, we have successfully developed an efficient in vitro micropropagation protocol for mass production. Nodal segments containing axillary buds from healthy female mother plants were used as explants. The objective of the present research was to investigate the in vitro explant disinfestation, culture establishment, shoot proliferation and root induction as well as the acclimatization of the in vitro micropropagated plantlets.

It is critical that the propagated clones maintain their pharmaceutical content production as high as the selected mother plants. Thus, in order to control the consistency of the minor phytocannabinoids production (CBD and CBG) between the female mother plants and their acclimatized in vitro propagated clones, at the harvest stage, quantitative nuclear magnetic resonance (qNMR) was used. Quantitative nuclear magnetic resonance is a modern analytical methodology with continuously increasing applications in complex mixtures permitting fast quantitation without having to separate analytes and without the need of standards [30,31,32], characteristics that make qNMR advantageous over techniques such as high-performance liquid chromatography (HPLC) and gas chromatography–mass spectrometry (GC-MS) [33]. Furthermore, CBD and CBG content were evaluated in different developmental stages.

## 2. Results

The disinfestation protocol of *Cannabis* explants was absolute effective. Shoot and root formation and growth were significantly affected, depending on the type and concentration of plant growth regulators (Table 1 and Table 2, please see Figures S1 to S6 in Supplementary Materials). The effect of medium strength and plant growth regulators’ concentrations on the average number and length of shoots, shoot formation frequency, as well as on the average number and length of roots and root formation frequency of the two high CBD and CBG *Cannabis sativa* varieties’ explants are presented in Table 1 and Table 2 respectively.

### 2.1. Shoot Multiplication, Elongation and Regeneration

Shoot elongation was observed during the second week of culture while shoot proliferation was achieved after at least three weeks, depending on treatment. Within each variety, statistically significant differences (*p* ≤ 0.05) in average number and length of shoots and shoot formation frequency among different treatments were observed. For the high CBD cannabis variety, full-strength MS medium clearly outweighed half-strength medium in terms of shoots, although there was no statistical difference between the two treatments. Regarding the high CBD variety, MS 1X medium showed higher average shoot number (1.79) and length (2.42 cm) per explant as well as shooting percentage (58.33%) compared to MS 1/2X (1.42, 2.09 cm and 41.67% respectively). For the high CBG cannabis variety, the results were varied. Full-strength MS showed only higher average shoot number per explant (1.54) compared to half-strength (1.46). On the contrary, average shoot length was higher using 1/2X MS (2.44 cm) than 1X MS (2.14 cm). The rooting percentage was equal in both treatments (Table 1 and Table 2). There was no statistically difference between the two treatments respecting the measured traits. Full- and half-strength MS medium with no plant growth regulators presented the lowest average number and length of shoots per explant and shooting percentage for both varieties among all treatments (Table 1 and Table 2).

The cytokinin growth regulator type (BA and TDZ) and their concentrations had significant effect on the average number and length of shoots and shoot formation frequency for both varieties (Table 1 and Table 2). Overall, the highest average number and length of shoots were obtained when both full- and half-strength MS medium were supplemented with 4.0 μM BA. Regarding the high CBD variety, the highest average number and length of shoots in full-strength medium were 3.63 cm and 5.66 cm respectively, as in half-strength medium were 3.13 cm and 5.09 cm respectively. However, supplementing both media with 4.0 μM TDZ presented the second highest results although having no statistically significant difference with the optimum ones. For the high CBD variety, the highest shoot formation frequency (100%) was achieved when full MS supplemented with 8.0 μM BA, although there was no significant difference when 4.0 μM BA (95.83%) or 8.0 μM TDZ (95.83%) was used. In half-strength MS, the highest shoot percentage (91.67%) was obtained when it was supplemented with 4.0 μM BA or 8.0 μM TDZ. Concerning the high CBG variety, the best results in full-strength MS+4.0 μM BA were 3.38 shoots per explant with an average length of 6.23 cm, while the relative in half-strength medium were 3.08 shoots with an average length of 5.99 cm. TDZ in a concentration of 4.0 μM resulted somehow in lower shoots per explant and shoots average length values which apparently had no significant differences with previous treatments. The best shoot formation frequency for the high CBG variety (100%) concerning full MS, was achieved when it was supplemented with 2.0 μM BA or 8.0 μM TDZ, although it showed no significant difference when 4.0 μM BA was used (95.83%). Half-strength MS supplemented with 4.0 μM BA presented the highest shooting percentage (100%), and the second highest percentage (91.67%) with no significant difference at MS+2.0 or 4.0 μM TDZ.

### 2.2. Rooting of Shoots In Vitro

Root initiation of well-developed in vitro propagated shoots started during the second or the third week of culture media depending on rooting treatment. Within each variety, statistically significant differences (*p* ≤ 0.05) in average number and length of roots and root formation frequency among different treatments were observed. Moreover, the results concerning roots for both, high CBD and CBG cannabis varieties using MS medium with no plant growth regulators were contrary to the shoot traits. Half-strength MS medium clearly outweighed over full-strength regarding roots, although there was no statistically difference between the two treatments. For the high CBD variety, the best average number (1.17) and length (1.26 cm) of roots and root formation frequency (66.67%) was achieved in 1/2X MS medium compared to full-strength MS (0.96, 0.92 cm and 45.83% respectively). Concerning the high CBG variety, in 1/2X MS, the average number (1.00) and length (1.10 cm) of roots as well as root formation frequency (66.67%) was higher compared to full-strength MS (0.83, 0.76 cm and 45.83% respectively). Generally, free hormone full and half-strength MS medium presented the lowest average number and length of root per shoot as well as rooting percentage for both varieties among all treatments (Table 1 and Table 2). However, when half-strength MS was supplemented with 1 μM IBA that led to the lowest average root length per shoot (1.11 cm) and root formation frequency (54.17%) for the CBD variety and, 0.90 cm and 50.00% for CBG variety respectively.

Auxins (IBA and NAA) and their concentrations significantly influenced average number and length of roots and root formation frequency for both varieties (Table 1 and Table 2). The highest average root numbers per shoot, 3.17 and 3.04, were obtained for the CBD variety with the addition of 4.0 μM IBA in the 1X and 1/2X MS medium respectively, while the latter treatment presented the longest average roots (2.10 cm). When full-strength MS supplemented with 4.0 μM NAA, it showed the highest average root length (2.27 cm) concerning the CBD variety plants. For the same variety, the addition of 2.0 or 4.0 μM IBA in both half- and full-strength MS media resulted in the highest root formation frequency (87.50%). Half-strength MS+4.0 μM NAA treatment was no significant different to latter treatments concerning root formation frequency (83.33%) in the CBD variety plants.

Regarding the CBG variety, the highest average root numbers per shoot, 2.88 and 2.75, were achieved when 1X MS supplemented with 4.0 μM IBA and 1/2X MS with 4.0 μM NAA respectively. When both full- and half-strength MS supplemented with 4.0 μM NAA, the longest average roots were obtained, 1.88 cm and 1.85 cm respectively. For the same variety, the addition of 4.0 μM IBA in both half- and full-strength MS media or the addition of 2.0 μM IBA in half-strength MS resulted in the highest root formation frequency (87.50%).

### 2.3. Acclimatization

Rooted plantlets were successfully transplanted in plastic pots containing a 3 peat:1 pearlite (*v*/*v*) sterile mixture that was placed in mini greenhouses. The plantlets were easily acclimatized to ex vitro conditions with low necroses and within 2 weeks, new growth was observed. After two more weeks, the acclimatized plantlets were transplanted to flowerpots and placed indoors under controlled environmental conditions. These plants exhibited 96% survival rate. The acclimatized plants exhibited normal development with functional leaves and no morphological abnormalities which made easier to final plantlet acclimatization to the external environment. Eventually, these plants exhibited an overall 92% survival rate.

### 2.4. Chemical Analysis

**Figure 1:**
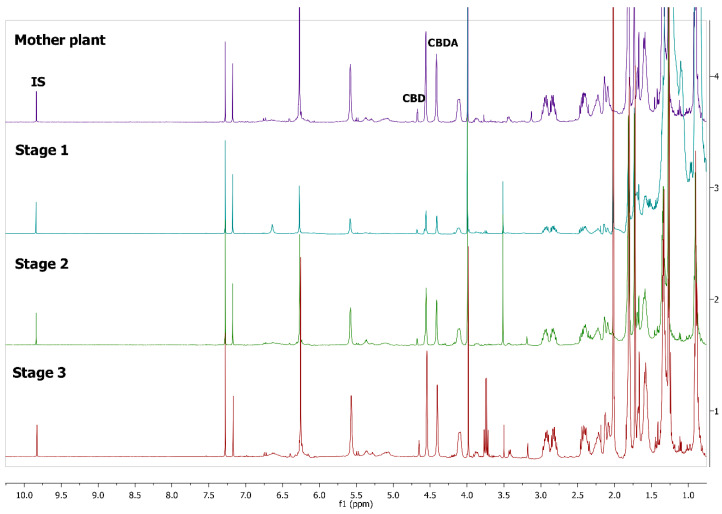
^1^Η-NMR spectra of mother plant with high concentration in cannabidiolic acid (CBDA) (top) and in vitro cultivated plants in three stages of growth.

**Figure 2:**
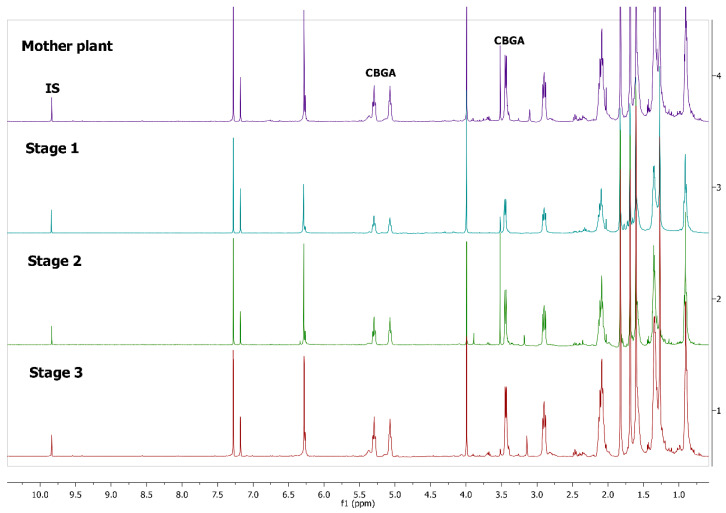
^1^Η-NMR spectra of mother plant with high concentration in CBGA (top) and in vitro cultivated plants in three stages of growth.

^1^H-NMR spectra of *Cannabis sativa* extracts, showing the characteristic peaks of the studied cannabinoids and internal standard in mature female plants (mother plants) as well as in mature acclimatized in vitro micropropagated plants in three stages of growth, are presented in Figure 1 and Figure 2. In each variety, the mature acclimatized in vitro micropropagated plants were randomly selected from all in vitro treatments.

**Figure 3:**
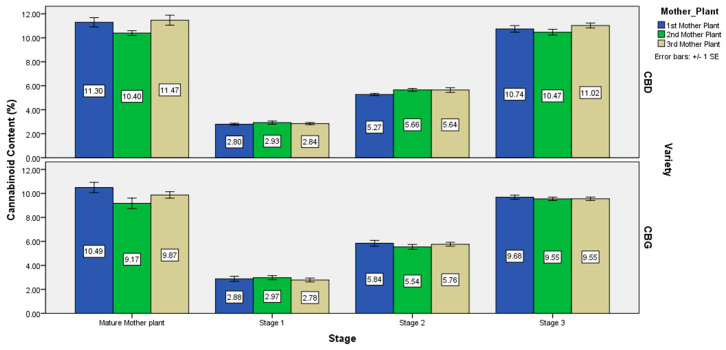
The CBD+CBDA and CBG+CBGA content (%) of the field grown mother plants and their clones at three different growth stages of the two high CBD and CBG *Cannabis sativa* varieties.

The chemical profiles of flower samples taken from healthy, high yielding cannabidiol and cannabigerol respectively, mother plants were randomly selected from three different field plots as shown in Figure 3. The chemical profiles of mature flower samples taken from the fully acclimatized of in vitro cultured clones at three different growth stages are shown in Figure 3, too. Both CBD+CBDA and CBG+CBGA content of the clones from each mother plant was increasing as plant age was approaching maturity, reaching its highest level during harvesting stage (Figure 3, please see Table S1 in Supplementary Materials). The highest concentration of CBD+CBDA and CBG+CBGA content of acclimatized in vitro micropropagated plants was found at 11.41% and 10.01% respectively, during the third (harvest) stage i.e., 130-day-old plants since the time that they were first placed in in vitro conditions. The highest concentration of CBD+CBDA and CBG+CBGA content of mother plants was found at 11.47% and 10.49% respectively.

Comparison between mother plants and their clones, for each variety, concerning CBD+CBDA and CBG+CBGA content respectively, showed no statistically significant differences (*p* ≤ 0.05) thus indicating consistency of their chemical profile (Table 3). Among the mother plants and their clones for each variety, there were no statistically significant differences (*p* ≤ 0.05) in CBD+CBDA and CBG+CBGA content, thus indicating homogeneity of the plant material within each variety. All flower samples were screened for the Δ^9^-THC concentration, by a certified laboratory, and none of them exceeded the legal content limit of 0.2%.

## 3. Discussion

One of the major issues concerning the use of natural products is their inadequacy in sufficient quantities. Supply can therefore become a major problem that can be overcome only if appropriate sources can be identified [32]. The constantly increasing need for cannabinoids deriving from natural sources imposes the need to detect *Cannabis sativa* varieties rich in bioactive secondary metabolites. Thus, in vitro propagation techniques of *Cannabis* sp. varieties with desirable chemical profiles and growth, could produce abundant and uniform plant material for commercial use. Moreover, the fact that the method of healthy clones’ mass micropropagation is both efficient, economical and thus attractive to pharmaceutical industry, makes it a powerful technique.

### 3.1. Shoot Multiplication, Elongation and Regeneration

Adequate shoot organogenesis was obtained from nodal explants in MS nutrient media containing BA or TDZ. In general, both *Cannabis sativa* varieties responded better in full-strength MS than in half-strength MS medium. In accordance to the results of this study were those of Wan Nurul Hidayah et al. [34] in *Pogostemon cablin*, also known as patchouli. In overall treatments, the number of shoots obtained in full-strength medium was greater compared to half-strength medium. The findings were also supported by Kumar et al. [35] in *Litchi chinensis*. Shoot regeneration in *Harpagophytum procumbens* was proved to be greater in full-strength MS although there was no significant difference when half-strength medium was used [36]. Grigoriadou et al. [37] reported similar results for the one of the two pear cultivars while the other showed opposite results. On the contrary, in similar experiments Villamor [38] in *Zingiber officinale*, Fadel et al. [39] in *Mentha spicata* as well as Taheri et al. [40] in *Ziziphora persica* indicated decrease of shoot number and length with the dilution of MS basal medium. Likewise, increased MS strength resulted in a decrease of height and number of shoots in *Typhonium flagelliforme* according to Rezali et al. [41]. These differentiations of the medium strength effect are probably associated with particular components of the culture medium [39] and may vary among varieties and depend on the type and the physiological condition of the explants [42].

BA was found to be the most efficient cytokinin presenting the best multiplication rate, average shoot length and shooting percentage as well. Direct cannabis shoot proliferation was succeeded by Richez-Dumanois et al. [43], using the same cytokinin although at lower concentration, from apical and axillary bud explants. In this study, supplementing the nutrient media with TDZ, in same concentrations as BA, exhibited slightly lower values in shooting traits although both BAP and TDZ were individually effective in shoot formation and no significant differences between the respective treatments were observed. On the contrary, there are studies denoting TDZ efficiency in MS medium in inducing in vitro shoots over BA. Lata et al. [44,45] and Wang et al. [46] using nodal segments with axillary buds and shoot tips respectively for in vitro propagation, indicated a significant effect of TDZ on shoot formation in cannabis plantlets, which performed better than the relative of BA. Moreover, Lata et al. [47] have accomplished propagation through alginate encapsulation of axillary buds of *Cannabis sativa* and reported that encapsulated explants exhibited the best regrowth and conversion frequency on MS nutrient medium after being supplemented with TDZ. This highest response on cannabis shoot induction was achieved in much lower TDZ concentrations than the relatives of this research, although in their studies THC varieties were used. However, in the experiments of this study, higher concentrations of TDZ suppressed shoot formation and caused reduction in shoot length, results that were in accordance with those of Lata et al. [45,48] and Huetteman and Preece [49]. Unlike all the above results, Slusarkiewicz-Jarzina et al. [50] did not succeed to regenerate cannabis in adequate scale using internode explants probably due to the different plant growth regulators used.

### 3.2. Rooting of Shoots In Vitro

All the adventitious shoots from the previous shooting stage used in rooting phase produced roots in every treatment. The rooting percentages were significantly different among rooting treatments thus indicating the intricacy of root induction though in several species. Richez-Dumanois et al. [43] described that rooting was extremely difficult and its response was poor. Comparing rooting between full- and half-strength MS, the latter presented higher values for all measured traits. Other researchers have also reported the beneficial effect of the medium strength reduction on root initiation [51,52]. Rezali et al. [41] reported an increase in the number of roots in *Typhonium flagelliforme* when MS strength decreased. Bidarigh and Azarpour [53] also found that highest root length and root number in micro cuttings of tea (*Camellia sinensis*) were obtained by diminuting the nutrient medium strength. Halving the strength of MS medium resulted in increased rooting traits of *Mentha spicata* [39] and *Mentha arvensis* [54]. Root number in *Zingiber officinale* [38], rooting percentage and root number per shoot in *Syzygium alternifolium* [55] were enhanced with the dilution of MS basal medium.

Rooting of shoot cultures were significantly influenced not only by the strength of MS medium but also by the addition of IBA and NAA. *Cannabis sativa* shoots rooted in all treatments in which different concentrations of IBA and NAA were used. The presence of IBA was found to be more efficient than NAA in average root number per shoot and rooting frequency. Best values in average number and length of roots in *Cannabis sativa* according to the literature were achieved using IBA [56]. Our results are in accordance with those of Lata et al. [44] too, although our correspondence rooting values are somehow lower. Furthermore, unlike both this and other studies, Lata et al. [44] reported root development in only ten days. On the contrary, this study observed the best average root length in NAA treatments while Lata et al. [44] recorded the relative ones in IBA treatments. In both studies the average root length values were similar. According to Movahedi et al. [57], the longest root was formed in MS medium containing NAA, too, while the highest root induction was reported in treatments with low IBA concentration. Once more, the presence of IBA was found to lead in significantly higher rooting traits in *Cannabis sativa* [48,58]. This promoting effect of IBA on the in vitro rooting of shoots has also been reported in several medicinal plants by Ferreira and Handro [59], Aminah et al. [60], Nadeem et al. [61], Zygomala et al. [62], Patel and Shah [52].

Conversely, Ślusarkiewicz-Jarzina et al. [50] reported root induction on MS basal medium containing IAA and NAA while Smýkalová et al. [63] achieved root induction on growth regulator-free or supplemented with NAA- media.

### 3.3. Acclimatization

In vitro rooted plantlets were successfully acclimatized, applying a two-phase acclimatization protocol, exhibiting high survival rate. New growth was observed within two weeks, unlike Lata et al. [45] who observed that new leaves started to appear after 30–40 days. Two-step acclimatization process was applied by Lata et al. [44] and Lata et al. [56], too. In both studies, rooted plantlets after 8 weeks exhibited a slightly higher survival rate than the one of this study. Chandra et al. [58] reported acclimatization of all plantelets after four weeks of growth in hardening conditions. According to Lata et al. [48] complete acclimatization of the plantlets was attributed to the remarkably superiority of meta-Topolin over IBA which led to thicker and robust roots with lot of branches. Movahedi et al. [57] achieved survival rates similar to these of the present study in seedlings produced via tissue culture. Ślusarkiewicz-Jarzina et al. [50] also reported successfully acclimatization of in vitro rooted plantlets derived from callus in several *Cannabis sativa* cultivars.

### 3.4. Chemical Analysis

The chemical profiles of the mature flowers between mother plants and their in vitro propagated plantlets of both high CBD and CBG varieties respectively, were found to be analogous, having no significant difference during the harvest period. Chandra et al. [58], using gas chromatography-flame ionization detection (GC-FID), concluded that chemical profiles of in vitro and vegetatively propagated of *Cannabis sativa* plants were found to be identical to each other and also to that of the mother plant. Similar results were recorded by Lata et al. [56] and Lata et al. [48] in high yielding THC *Cannabis sativa* plants. In addition, the results of Ma and Gang [64], who compared the metabolic profiling of micropropagated and conventionally greenhouse grown plants of *Zingiber officinale* (ginger), indicated that no significant differences existed between growth treatments, suggesting that the biochemical mechanisms are not affected by in vitro propagation. Moreover, Sahoo et al. [65] performing a comparative GC-MS analysis of essential oils, total phenolic and total flavonoid content between the in vitro propagated and conventionally propagated plants of galanga (*Alpinia galanga*) showed no significant differences in phytoconstituents. Furthermore, it was seen that the total phenolic and total flavonoid content values slightly increased in micropropagated plants [65]. These findings are in accordance with the results of Behera et al. [66] and Bhardwaj et al. [67] on *Hedychium coronarium* and *Rhodiola imbricate* respectively.

Although few minor differences in cannabidiol and cannabigerol content in each variety respectively were observed among mother plants, there was homogeneity in their chemical profile. This homogeneity was also observed in the clones of mother plants showing high level chemical consistency in terms of secondary metabolites production. Moreover, both mother and in vitro micropropagated plants followed the same trend in respective cannabinoids concentration during plant growth and development. Both characteristics, homogeneity and trend in cannabinoids content of the micropropagated plantlets, were observed by Lata et al. [56], Chandra et al. [58] and Lata et al. [48].

In conclusion, the current study demonstrated an efficient micropropagation protocol of selected high CBD and CBG *Cannabis sativa* varieties. Moreover, the produced acclimatized in vitro cultured plants presented a consistent chemical profile comparable to the conventionally grown mother plants. Future research of this developed micropropagation protocol, potentially applicable in industrial scale agriculture, includes breeding program focusing on the improvement of high cannabinoids content varieties through selection.

## 4. Materials and Methods

### 4.1. Plant Material—Explants Disinfestation—Culture Establishment

Two varieties, a high CBD and a high CBG, of *Cannabis sativa* L. (Cannabaceae) were included in the present study, and kindly provided by Ekati Alchemy Lab SL (Barcelona, Spain). Establishment of shoot cultures were initiated using as explants nodal segments (1.0–1.5 cm long) containing one axillary bud each. Explants were excised from selected CBD and CBG enriched healthy young mother plants, at vegetative growth stage, grown in the greenhouse at the Institute of Mediterranean and Forest Ecosystems of the Hellenic Agricultural Organization “Demeter”. Only elite, based on chemical profile, female plants were used in the experiments. The mother plants were maintained at the vegetative stage under a photoperiod of 18 h until in vitro shoot cultures were established. All the plants were kept indoor, under controlled environmental conditions at 27 ± 2 °C with a 12-h fluorescent photoperiod, having approximately 500 μmol m^−2^ s^−1^ photosynthetic photon flux density, from flowering until maturity. Flower samples of mature female plants were collected and analyzed for their CBD and CBG concentration using qNMR.

Explants surface disinfestation was obtained by successive immersions in two different aqueous solutions: the first aqueous solution of sodium hypochlorite (10% NaOCl, Fluka, Germany) at concentrations 1.0% (*v*/*v*), supplemented with 0.05% (*v*/*v*) Tween-20 (Fisher Bioreagents, Pittsburgh, PA, USA), for 15 min with continuous stirring, and the second of 70% ethanol for 1 min. Each immersion was followed by three rinses with sterile deionized water for three minutes each.

Each explant was placed in a 25 mm × 150 mm glass culture tube, containing 20 mL of MS culture medium [68], supplemented with 4 and 8 μM of 6-benzyl-amino-purine (BA) (Sigma Chemicals, Saint Louis, MO, USA), 3% (*w*/*v*) sucrose (Duchefa Biochemie, Haarlem, The Netherlands), and 0.6% (*w*/*v*) agar (Duchefa Biochemie, Haarlem, The Netherlands). The pH of the medium was adjusted to 5.8 with 0.1 N NaOH or 0.1 N HCl prior to agar addition. The medium, the culture tubes as well as all equipment used in in vitro operations under aseptic conditions were sterilized by autoclaving (121 °C, 122 kPa) for 20 min. All cultures were incubated in a growth chamber at 23 ± 1 °C with 16 h photoperiod, under cool-white fluorescent lamps of 50 μmol m^−2^ s^−1^ photosynthetic photon flux density at culture level.

### 4.2. Shoot Multiplication, Elongation and Regeneration

After 30 days, the healthy explants without contamination, produced from the previously culture step, were subcultured in full- or half-strength MS medium (Murashige and Skoog 1962), supplemented with 6-benzylaminopurine (BA) (Sigma Chemicals, Saint Louis, MO, USA) or thidiazuron (TDZ) (Cayman Chemicals, Ann Arbor, MI, USA) at various concentrations (1.0, 2.0, 4.0 and 8.0 µM), 3% (*w*/*v*) sucrose, 0.6% (*w*/*v*) agar for multiple shoot induction in glass tubes. The pH of the medium was adjusted to 5.8 with 0.1N NaOH or 0.1N HCl prior to agar addition. The medium, the culture tubes as well as all the equipment used in in vitro operations under aseptic conditions were sterilized by autoclaving (121 °C, 122 kPa) for 20 min. After a 4-week period, the effect of the various concentrations of used plant growth regulators in relation to medium strength on shoot formation percentage (%), number and length of shoots were evaluated. Eight explants in three replications were used for each treatment. All cultures were incubated in a growth chamber at 23 ± 1 °C with 16 h photoperiod, under cool-white fluorescent lamps of 50 μmol m^−2^ s^−1^ photosynthetic photon flux density at culture level. Each experiment was arranged in the growth chamber in a completely randomized design.

### 4.3. Rooting of Shoots In Vitro

Shoots, 2.5–3.0 cm long, with well-developed leaves, derived from shoot multiplication, elongation and regeneration step, were transferred under aseptic conditions on glass tubes containing full- or half-strength MS for rooting. The nutrient medium was solidified by 0.7% (*w*/*v*) agar and supplemented with 1.0, 2.0, 4.0 or 8.0 μM of indole-3-butyric acid (IBA) (Sigma Chemicals, Saint Louis, MO, USA) or α-naphthalene acetic acid (NAA) (Sigma Chemicals, Saint Louis, MO, USA). The cultures were maintained in a growth chamber under the same conditions mentioned above. After a 4-week period, the effect of the various concentrations of the used plant growth regulators in relation to medium strength on the rooting percentage (%), the number and length of roots per shoot were evaluated. Eight shoots in three replications were used for each treatment and the experiments were arranged in the growth chamber in a completely randomized design.

### 4.4. Acclimatization

Plantlets with well-developed roots, were transplanted in 6.5 cm × 6.5 cm × 8.0 cm plastic pots containing a 3 peat:1 pearlite (*v*/*v*) sterile mixture, after washing the roots to remove agar. The pots were placed in 48cm × 33cm × 20cm mini greenhouses (Nortene, Ballée, France) with plastic cover in order to avoid water loss and maintain humidity. All the plantlets were kept under controlled environmental conditions at 27 ± 2 °C with a 16 h fluorescent photoperiod, with an approximately 50 μmol m^−2^ s^−1^ photosynthetic photon flux density. The plantlets were irrigated individually every day, if necessary, with tap water, to maintain adequate moisture. Acclimatization was achieved by opening gradually the plastic cover. The acclimatized plantlets were transplanted to flowerpots and placed indoor, under controlled environmental conditions at 27 ± 2 °C with a 12-h fluorescent photoperiod, with an approximately 500 μmol m^−2^ s^−1^ photosynthetic photon flux density, from flowering until maturity, as mother plants. Flower samples of mature acclimatized in vitro propagated plants were collected and analyzed for their CBD and CBG concentrations using qNMR in order to be compared with the female mother plants they originally derived from.

### 4.5. Chemical Analysis

Flower samples taken from the three mature female plants (mother plants) were collected and analyzed for their cannabidiol (CBD) and cannabidiolic acid (CBDA) and cannabigerol (CBG) and cannabigerolic acid (CBGA) concentrations. Mature flower samples taken from three acclimatized in vitro cultured plants derived from each of the three mother plants, were analyzed for their CBD and CBDA and CBG and CBGA content. Chemical profile of the mother as well as the acclimatized in vitro cultured plants were compared to assess the CBD and CBDA and CBG and CBGA content consistency. Triplicate of each sample were used for the analysis of cannabidiol and cannabidiolic acid and cannabigerol and cannabigerolic acid using qNMR.

All samples were dried at 60 °C till constant weight and were kept in an excicator. The flower samples were milled and were placed overnight in a deep freezer (−76 °C). A second freeze-drying step took place for 12h at −52 °C and 0.03 mbar pressure to ensure almost complete removal of moisture and volatile compounds. Then, 100 mg (±0.1 mg) of the dried ground flower sample was weighed and placed in 15 mL Falcon plastic tubes and 10 mL 90:10% methanol: chloroform mixture (Panreac for analysis) was added. The tubes were transferred to an ultrasonic bath (Semat, St Albans, UK) for 15 min to complete the extraction. The samples were centrifuged at 3075× *g* for 15 min (Eppendorf 5810R, Hamburg, Germany). 10 mL of the clear supernatant carefully removed and transferred to 50 mL round-bottom flasks, where they were mixed with 1 mL of a syringaldehyde (Acros Organics) solution (0.5 mg/mL) in acetonitrile (Scharlau) (Internal standard, I.S.) and the mixture was evaporated in a vacuum rotary evaporator (Buchi, Flawil, Switzerland).

The extract of each sample obtained from the above-described procedure was dissolved in 750 μL deuterated chloroform (CDCl_3_) (Euriso-Top) and the solution was transferred to a 5 mm NMR tube. Each sample was analyzed in triplicate using a standard 90-degree excitation pulse, with a pulse width of 10 s and a prescan delay of 6.5 s. All measurements were performed at 298 K. Typically, 16 scans were collected into 32 K data points over a spectral width of 0–13 ppm (5263.18 Hz) with a relaxation delay of 10 s, an acquisition time of 3.11 s and a FID resolution of 0.32 Hz. The appropriate relaxation delay was determined by gradual increases (1, 2, 5, 8, 10, 15, 20 s until the ratio between the integration of the peak of internal standard and the peak of the target compounds remained unchanged). The matching, tuning, shimming, receiver gain adjustment as well as phasing and baseline correction were always first performed automatically and then manually to achieve the best result. Prior to Fourier transformation (FT), an exponential weighting factor corresponding to a line broadening of 0.3 Hz was applied. For the peaks of interest, accurate integration was performed manually. The concentration of cannabinoids was measured by comparing the area of the selected signal of Table 4 with that of the internal standard (IS) at 9.81 ppm, which was set as 1. The calculation of the concentration of total cannabinoids in mg/100 mg of dry material was performed using the following formula:

n_CB_ = n_is_ × a × I_CB_/I_is_ (1)

m_CB_ = n_CB_/MW_CB_ (2)

where n_is_ = 0.0027 mmol, I_is_ = 1, a = 1 for CBD and CBDA, a = 0.5 for CBG and CBGA, n_CB_ are the moles of cannabinoids and MW_CB_ is molecular weight of cannabinoids, as shown in Table 4.

The identity of all compounds was defined by literature data [69]. Non overlapping, undoubtedly defined peaks of protons were selected for quantitation. CBD was distinguished by the singlet of H-10 trans proton at 4.67 ppm, while CBDA by the H-10 cis proton at 4.41 ppm (Figure 1) as previously reported [70]. CBG and CBGA were quantified as a total based on the peak at 3.43–3.45 ppm which corresponds to H-1′a and H-1′b protons. Due to the very low concentrations of CBG, the molecular weight of CBGA was used for the expression of the results.

### 4.6. Statistical Analysis

Analysis of variance and Duncan’s multiple range test at *p* ≤ 0.05 were performed on the growth percentage in height [(initial height-terminal height)/initial height], the number of shoots per explant, the average length of shoots, the percentage of rooted microcuttings, the number of roots per explant, the average length of roots per treatment. Data in percentages were subjected to appropriate log or arcsine transformation for proportions before statistical analysis and were transformed back to percentages for presentation in Tables and Graphs. All statistical analysis was performed using SPSS v.20 software for Windows (IBM SPSS Statistics 2011, IBM Corp., Armonk, NY, USA).

## 5. Conclusions

An efficient in vitro micropropagation protocol was developed for the large-scale production of the two selected high CBD and CBG *Cannabis sativa* varieties. The regeneration method demonstrated high survival rate of rooted plantlets as well as high frequency of shoot formation and multiplication and root induction. The applied regeneration protocol of *Cannabis sativa* plants is useful for the conservation and mass propagation of the selected varieties. Their desirable chemical profiles, when applied in pharmaceutical industry denote once again their high added value. According to this study, any variation in shoot formation and root induction among the treatments of the plant material may be influenced by the cultivar, indicating that these differences are genotype dependent. The chemical profiles of conventionally grown mother plants and their in vitro propagated clones of selected *Cannabis sativa* varieties were found to be identical to each other, indicating that the biochemical mechanisms of CBD and CBG production are not affected by in vitro propagation techniques. In conclusion, the developed propagation protocol suggest potential mass production of high yielding CBD and CBG varieties of *Cannabis sativa* plants applied in the pharmaceutical industry.

## Figures and Tables

**Table 1:** The effect of medium strength and plant growth regulators’ concentrations on the average number and length of shoots per explant, shoot formation frequency, as well as on number and length of roots per shoot and rooting percentage of the high cannabidiol (CBD) *Cannabis sativa* variety. (Means followed by the same letter do not differ statistically at *p* ≤ 0.05 according to Duncan test).

Medium Strength	Concentration (μΜ)	Average Number of Shoots per Explant	Average Shoot Length (cm)	Average Shooting Percentage (%)
MS 1X	No PGR	1.79 ^ijk^	2.42 ^hi^	58.33 ^cd^
	1.0 μM BA	2.08 ^ghi^	2.48 ^ghi^	79.17 ^abc^
	2.0 μM BA	2.67 ^cde^	3.98 ^cd^	87.50 ^a^
	4.0 μM BA	3.63 ^a^	5.66 ^a^	95.83 ^a^
	8.0 μM BA	2.71 ^cd^	3.67 ^de^	100.00 ^a^
	1.0 μM TDZ	2.21 ^efghi^	2.87 ^fgh^	87.50 ^a^
	2.0 μM TDZ	2.83 ^cd^	3.22 ^defg^	100.00 ^a^
	4.0 μM TDZ	3.29 ^ab^	5.38 ^a^	95.83 ^a^
	8.0 μM TDZ	2.13 ^fghi^	3.73 ^de^	91.67 ^a^
MS 1/2X	No PGR	1.42 ^k^	2.09 ^i^	41.67 ^d^
	1.0 μM BA	1.58 ^jk^	2.47 ^ghi^	54.17 ^d^
	2.0 μM BA	2.42 ^defgh^	4.50 ^bc^	87.50 ^a^
	4.0 μM BA	3.13 ^bc^	5.09 ^ab^	91.67 ^a^
	8.0 μM BA	2.38 ^defgh^	3.68 ^de^	87.50 ^a^
	1.0 μM TDZ	1.75 ^ijk^	2.53 ^fghi^	62.50 ^bcd^
	2.0 μM TDZ	2.50 ^defg^	3.19 ^efg^	83.33 ^ab^
	4.0 μM TDZ	2.58 ^def^	4.97 ^ab^	91.67 ^a^
	8.0 μM TDZ	1.96 ^hij^	3.25 ^def^	79.17 ^abc^
MS 1X	No PGR	0.96 ^g^	0.92 ^g^	45.83 ^c^
	1.0 μM IBA	1.13 ^fg^	1.12 ^efg^	54.17 ^bc^
	2.0 μM IBA	3.13 ^a^	1.93 ^abc^	87.50 ^a^
	4.0 μM IBA	3.17 ^a^	1.87 ^abcd^	87.50 ^a^
	8.0 μM IBA	2.13 ^bcde^	1.54 ^bcdefg^	66.67 ^abc^
	1.0 μM NAA	1.29 ^efg^	1.02 ^fg^	54.17 ^bc^
	2.0 μM NAA	1.46 ^efg^	1.15 ^efg^	62.50 ^abc^
	4.0 μM NAA	2.92 ^abc^	2.27 ^a^	83.33 ^ab^
	8.0 μM NAA	2.04 ^cdef^	1.88 ^abcd^	75.00 ^abc^
MS 1/2X	No PGR	1.17 ^fg^	1.26 ^defg^	66.67 ^abc^
	1.0 μM IBA	1.42 ^efg^	1.11 ^efg^	54.17 ^bc^
	2.0 μM IBA	2.83 ^abc^	1.74 ^abcde^	87.50 ^a^
	4.0 μM IBA	2.96 ^ab^	1.67 ^abcdef^	87.50 ^a^
	8.0 μM IBA	1.88 ^defg^	1.43 ^cdefg^	66.67 ^abc^
	1.0 μM NAA	1.33 ^efg^	1.08 ^fg^	62.50 ^abc^
	2.0 μM NAA	1.75 ^efg^	1.34 ^cdefg^	70.83 ^abc^
	4.0 μM NAA	3.04 ^a^	2.10 ^ab^	83.33 ^ab^
	8.0 μM NAA	2.67 ^abcd^	1.98 ^abc^	83.33 ^ab^

No PGR: no plant growth regulator, BA: 6-benzylaminopurine, TDZ: thidiazuron, IBA: indole-3-butyric acid, NAA: α-naphthalene acetic acid. Means followed by the same letter do not differ statistically at *p* ≤ 0.05 according to Duncan test.

**Table 2:** The effect of medium strength and plant growth regulators’ concentrations on the average number and length of shoots per explant, shoot formation frequency, as well as on number and length of roots per shoot and rooting percentage of the high cannabigerol (CBG) *Cannabis sativa* variety. (Means followed by the same letter do not differ statistically at *p* ≤ 0.05 according to Duncan test).

Medium Strength	Concentration (μΜ)	Average Number of Shoots per Explant	Average Shoot Length (cm)	Average Shooting Percentage (%)
MS 1X	No PGR	1.54 ^hij^	2.14 ^i^	45.83 ^d^
	1.0 μM BA	1.88 ^fghij^	3.34 ^fgh^	75.00 ^abc^
	2.0 μM BA	2.79 ^bc^	4.65 ^cd^	100.00 ^a^
	4.0 μM BA	3.38 ^a^	6.23 ^a^	95.83 ^a^
	8.0 μM BA	2.46 ^cde^	4.32 ^cde^	91.67 ^a^
	1.0 μM TDZ	1.71 ^ghij^	2.76 ^hi^	54.17 ^cd^
	2.0 μM TDZ	2.54 ^cd^	3.93 ^def^	95.83 ^a^
	4.0 μM TDZ	3.21 ^a^	5.97 ^ab^	100.00 ^a^
	8.0 μM TDZ	1.96 ^fghi^	4.25 ^def^	83.33 ^ab^
MS 1/2X	No PGR	1.46 ^j^	2.44 ^hi^	45.83 ^d^
	1.0 μM BA	1.67 ^hij^	3.00 ^ghi^	66.67 ^bcd^
	2.0 μM BA	2.17 ^def^	5.15 ^bc^	79.17 ^ab^
	4.0 μM BA	3.08 ^ab^	5.99 ^ab^	100.00 ^a^
	8.0 μM BA	2.04 ^efgh^	4.03 ^def^	79.17 ^ab^
	1.0 μM TDZ	1.50 ^j^	2.68 ^hi^	50.00 ^d^
	2.0 μM TDZ	2.13 ^defg^	3.97 ^def^	91.67 ^a^
	4.0 μM TDZ	2.42 ^cde^	5.67 ^ab^	91.67 ^a^
	8.0 μM TDZ	1.75 ^fghij^	3.66 ^efg^	75.00 ^abc^
MS 1X	No PGR	0.83 ^g^	0.76 ^g^	45.83 ^c^
	1.0 μM IBA	0.96 ^fg^	0.97 ^fg^	54.17 ^bc^
	2.0 μM IBA	2.71 ^a^	1.67 ^abc^	87.50 ^a^
	4.0 μM IBA	2.88 ^a^	1.63 ^abcd^	87.50 ^a^
	8.0 μM IBA	1.92 ^bcd^	1.35 ^bcdefg^	70.83 ^abc^
	1.0 μM NAA	1.17 ^defg^	0.92 ^fg^	62.50 ^abc^
	2.0 μM NAA	1.21 ^defg^	1.01 ^efg^	62.50 ^abc^
	4.0 μM NAA	2.79 ^a^	1.88 ^a^	87.50 ^a^
	8.0 μM NAA	1.75 ^cdef^	1.54 ^abcde^	75.00 ^abc^
MS 1/2X	No PGR	1.00 ^fg^	1.10 ^defg^	66.67 ^abc^
	1.0 μM IBA	1.25 ^defg^	0.90 ^fg^	50.00 ^c^
	2.0 μM IBA	2.50 ^abc^	1.59 ^abcd^	87.50 ^a^
	4.0 μM IBA	2.67 ^ab^	1.54 ^abcde^	87.50 ^a^
	8.0 μM IBA	1.83 ^cde^	1.38 ^abcdef^	70.83 ^abc^
	1.0 μM NAA	1.08 ^efg^	1.00 ^efg^	62.50 ^abc^
	2.0 μM NAA	1.50 ^defg^	1.24 ^cdefg^	66.67 ^abc^
	4.0 μM NAA	2.75 ^a^	1.85 ^ab^	83.33 ^ab^
	8.0 μM NAA	2.42 ^abc^	1.78 ^abc^	83.33 ^ab^

No PGR: no plant growth regulator, BA: 6-benzylaminopurine, TDZ: thidiazuron, IBA: indole-3-butyric acid, NAA: α-naphthalene acetic acid. Means followed by the same letter do not differ statistically at *p* ≤ 0.05 according to Duncan test.

**Table 3:** Analysis of variance of the CBD+CBDA and CBG+CBGA content (%) of the three field grown mother plants and their clones of the two high CBD and CBG *Cannabis sativa*. (Means followed by the same letter do not differ statistically at *p* = 0.05 according to Duncan test.).

CBD+CBDA (%)					CBG+CBGA (%)		
Mother Plant		1	2	3	1	2	3
		11.30 ^a^	10.40 ^a^	11.47 ^a^	10.49 ^b^	9.17 ^b^	9.87 ^b^
Clone	1	10.90 ^a^	10.28 ^a^	10.57 ^a^	10.01 ^b^	9.51 ^b^	9.52 ^b^
	2	10.22 ^a^	10.25 ^a^	11.09 ^a^	9.62 ^b^	9.75 ^b^	9.60 ^b^
	3	11.10 ^a^	10.88 ^a^	11.41 ^a^	9.41 ^b^	9.39 ^b^	9.55 ^b^
Clone Mean		10.74 ^a^	10.47 ^a^	11.02 ^a^	9.68 ^b^	9.55 ^b^	9.55 ^b^
Overall Mother Plant Mean		11.05 ^a^			9.84 ^b^		
Overall Clone Mean		10.74 ^a^			9.59 ^b^		

CBD: cannabidiol, CBDA: cannabidiolic acid, CBG: cannabigerol and CBGA: cannabigerolic acid. Means followed by the same letter do not differ statistically at *p* ≤ 0.05 according to Duncan test.

**Table 4:** Molecular weight, structure and selected signals (ppm) of cannabinoids.

Cannabinoids	MW	Structure	Proton Signal	δ in ppm
CBD	314.5	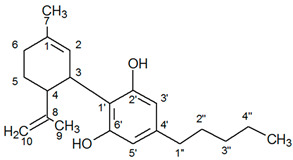	H-10 trans	4.67
			H-10 cis	4.57
			H-2	5.59
CBDA	358.5	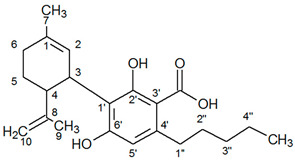	H-5′	6.27
			H-10 trans	4.55
			H-10 cis	4.41
			H-2	5.56
CBG	316.5	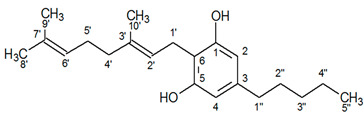	H-2/H-4	6.25
			H-1′a/H-1′b	3.43
			H-2′	5.29
			H-6′	5.07
CBGA	360.5	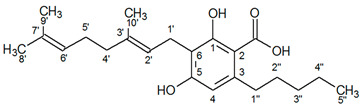	H-4	6.27
			H-1′a/H-1′b	3.45
			H-2′	5.30
			H-6′	5.07

## Supplementary Materials

The following are available online, Figure S1. The effect of medium strength and plant growth regulators’ concentration on the average number of shoots per explant of the high CBD and CBG *Cannabis sativa* varieties. Figure S2. The effect of medium strength and plant growth regulators’ concentration on the average length (cm) of shoots per explant of the high CBD and CBG *Cannabis sativa* varieties. Figure S3. The effect of medium strength and plant growth regulators’ concentration on the shoot formation percentage (%) of the high CBD and CBG *Cannabis sativa* varieties. Figure S4. The effect of medium strength and plant growth regulators’ concentration on the average number of roots per shoot of the high CBD and CBG *Cannabis sativa* varieties. Figure S5. The effect of medium strength and plant growth regulators’ concentration on the average length (cm) of roots per shoot of the high CBD and CBG *Cannabis sativa* varieties. Figure S6. The effect of medium strength and plant growth regulators’ concentration on the rooting. Figure S7. The CBD+CBDA and CBG+CBGA content (%) of the field grown mother plants and their clones of the two high CBD and CBG *Cannabis sativa* varieties. Table S1. CBD+CBDA and CBG+CBGA content (%) of the field grown mother plants and their clones at different developmental stages of the two high CBD and CBG *Cannabis sativa* varieties (Data represent average ± standard deviation).
