# Supplementary material for: Biotechnological Approaches on Two High CBD and CBG Cannabis sativa L. (Cannabaceae) Varieties: In Vitro Regeneration and Phytochemical Consistency Evaluation of Micropropagated Plants Using Quantitative 1H-NMR
Source: Molecules. 2020 Dec 15;25(24):5928. doi: 10.3390/molecules25245928 (PMC7765244; doi:10.3390/molecules25245928)
Supplement: Supplementary file 1 [file molecules-25-05928-s001.pdf]

molecules

MDPI

Article

Biotechnological approaches on two high CBD and CBG *Cannabis sativa* L. (Cannabaceae) varieties: In vitro regeneration and phytochemical consistency evaluation of micropropagated plants using quantitative ^{1}H-NMR

Kostas Ioannidis [1,*], Evangelos Dadiotis [2], Vangelis Mitsis [3], Eleni Melliou [2] and Prokopios Magiatis [2]

1 Laboratory of Sylviculture, Forest Genetics and Biotechnology, Institute of Mediterranean and Forest Ecosystems, Hellenic Agricultural Organization "Demeter", Ilissia, 11528 Athens, Greece
2 Department of Pharmacognosy and Natural Products Chemistry, Faculty of Pharmacy, National and Kapodistrian University of Athens, Panepistimiopolis Zografou, 15771 Athens, Greece; vaggdad@gmail.com (E.D.); emelliou@pharm.uoa.gr (E.M.); magiatis@pharm.uoa.gr (P.M.)
3 Ekati Alchemy Lab SL, 08180 Barcelona, Spain; ekatimed@gmail.com
* Correspondence: ioko@fria.gr; Tel.: +30-210-7783-750

Received: 12 December 2020; Accepted: 13 December 2020; Published: 15 December 2020

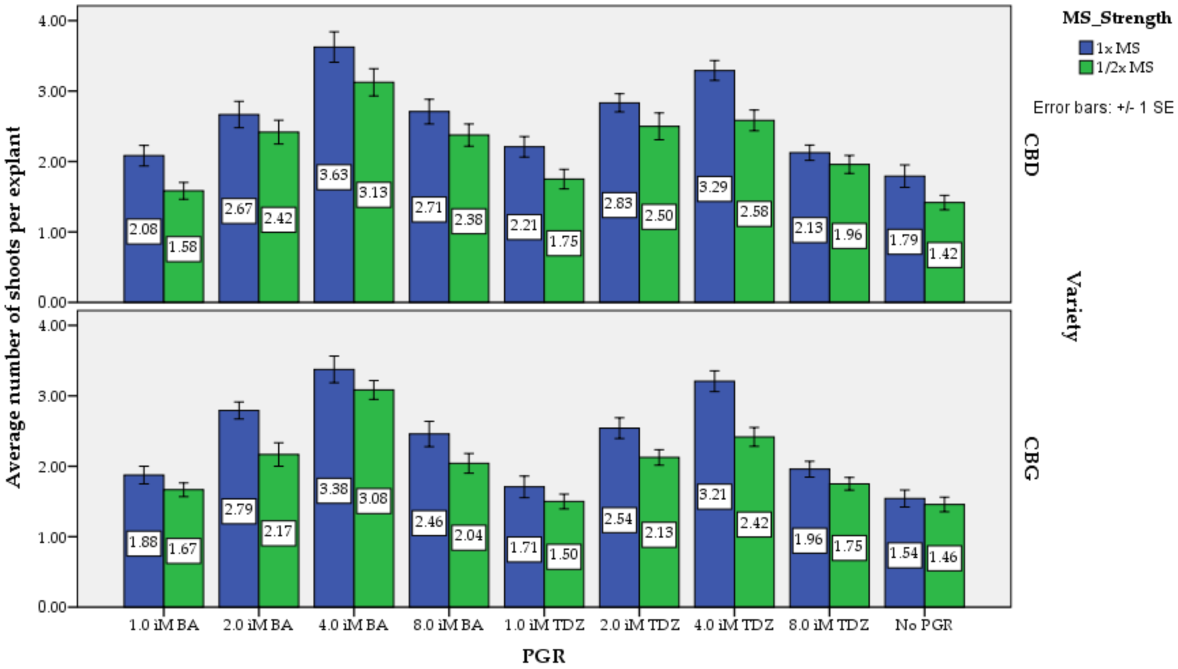

Figure S1. The effect of medium strength and plant growth regulators' concentration on the average number of shoots per explant of the high CBD and CBG *Cannabis sativa* varieties.

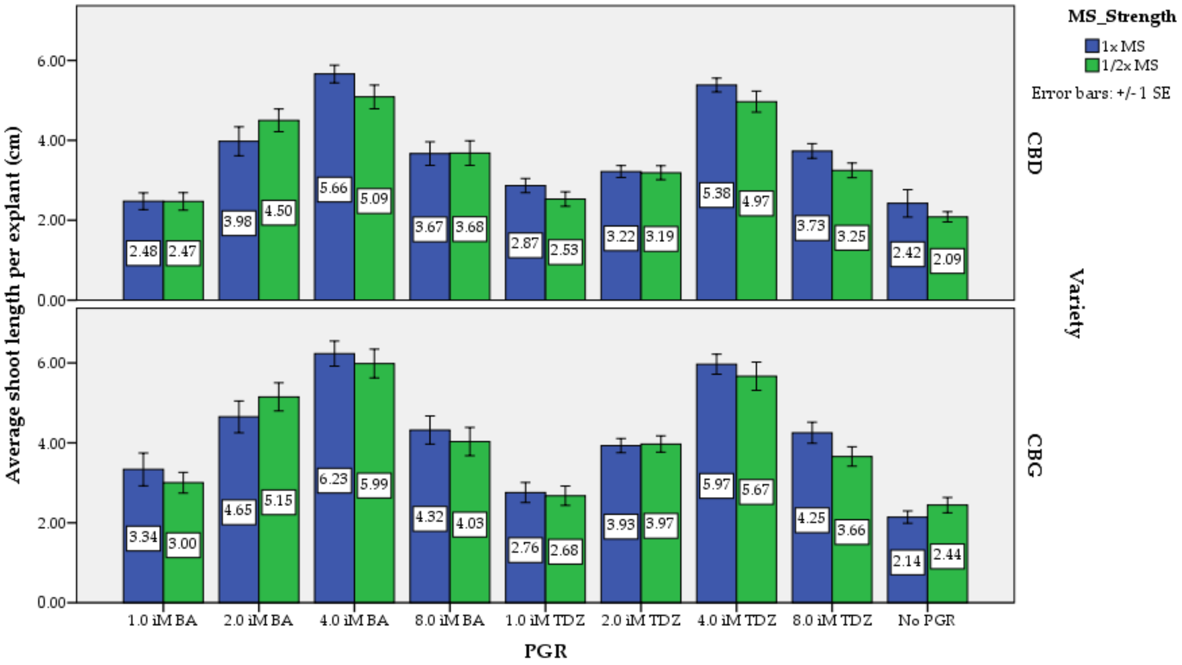

Figure S2. The effect of medium strength and plant growth regulators' concentration on the average length (cm) of shoots per explant of the high CBD and CBG *Cannabis sativa* varieties.

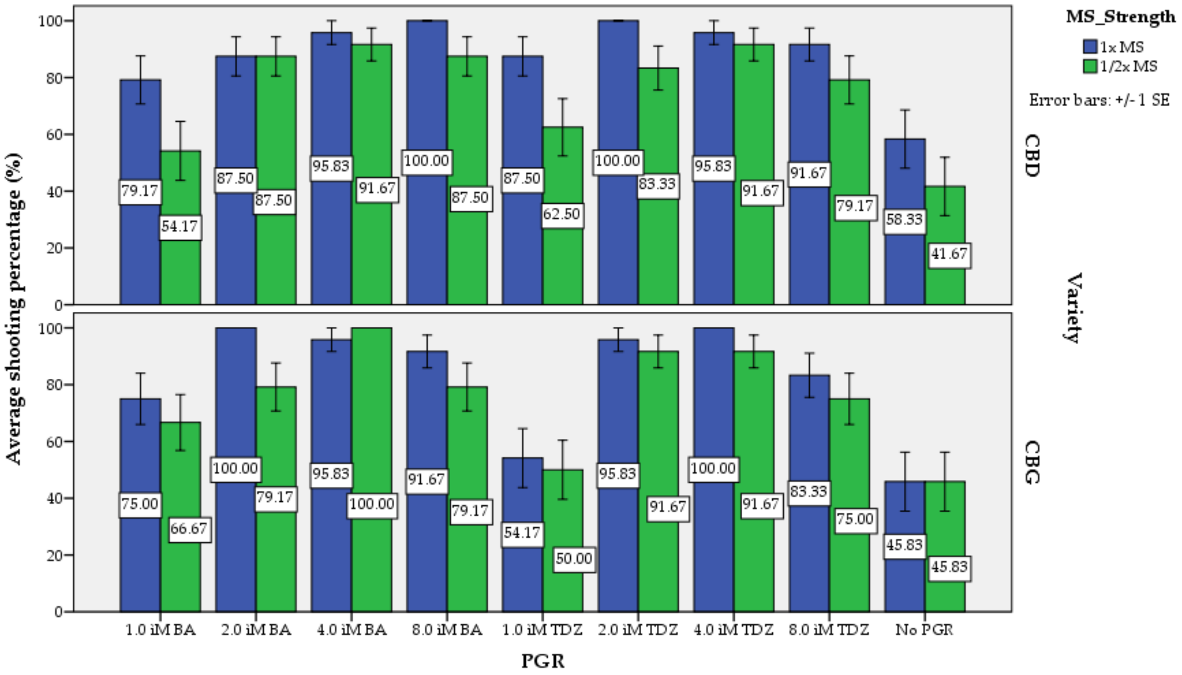

Figure S3. The effect of medium strength and plant growth regulators' concentration on the shoot formation percentage (%) of the high CBD and CBG *Cannabis sativa* varieties.

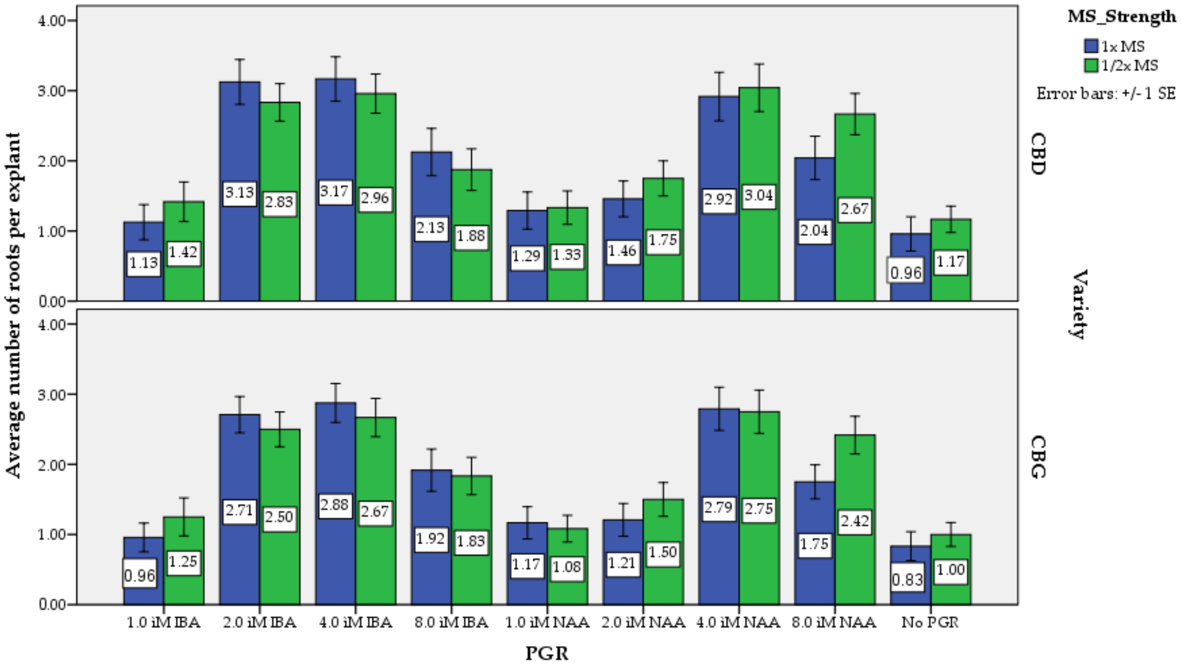

Figure S4. The effect of medium strength and plant growth regulators' concentration on the average number of roots per shoot of the high CBD and CBG *Cannabis sativa* varieties.

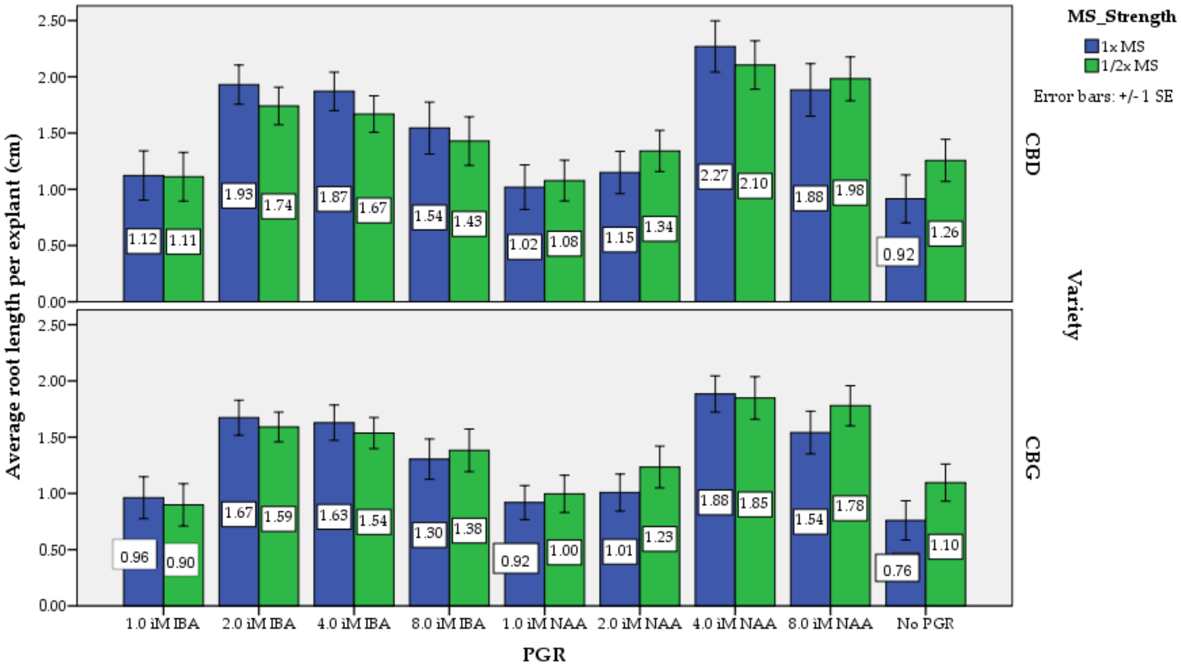

Figure S5. The effect of medium strength and plant growth regulators' concentration on the average length (cm) of roots per shoot of the high CBD and CBG *Cannabis sativa* varieties.

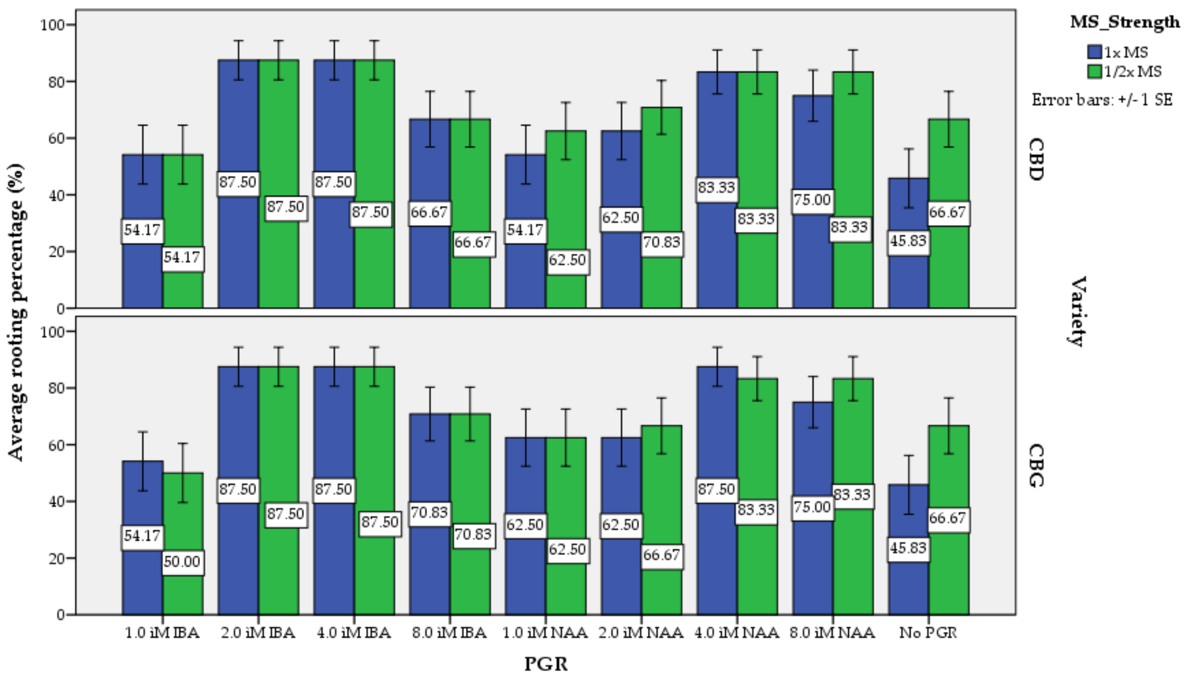

Figure S6. The effect of medium strength and plant growth regulators' concentration on the rooting.

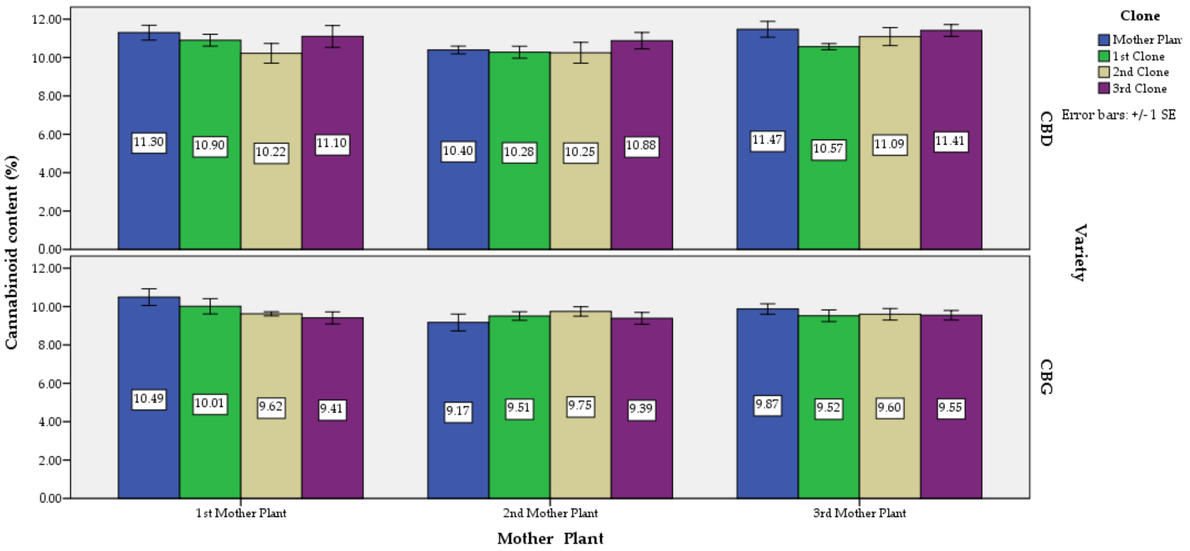

Figure S7. The CBD+CBDA and CBG+CBGA content (%) of the field grown mother plants and their clones of the two high CBD and CBG *Cannabis sativa* varieties.

Table S1. CBD+CBDA and CBG+CBGA content (%) of the field grown mother plants and their clones at different developmental stages of the two high CBD and CBG *Cannabis sativa* varieties (Data represent average ± standard deviation).

Variety	Mother Plant (%)	Clone	Stage 1 (%)	Stage 2 (%)	Stage 3 (%)	Clone Mean at Maturity
CBD + CBDA	11.30±0.67	1	2.71 ± 0.35	5.39 ± 0.11	10.90 ± 0.54	10.74 ± 0.46
		2	2.64 ± 0.06	5.18 ± 0.37	10.22 ± 0.89	
		3	3.04 ± 0.08	5.24 ± 0.28	11.10 ± 0.98	
	10.40 ± 0.35	1	2.99 ± 0.38	5.37 ± 0.45	10.28 ± 0.54	10.47 ± 0.36
		2	2.54 ± 0.24	5.86 ± 0.23	10.25 ± 0.94	
		3	3.25 ± 0.21	5.73 ± 0.27	10.88 ± 0.74	

	11.47 ± 0.72	1	3.00 ± 0.26	5.07 ± 0.30	10.57 ± 0.28	11.02 ± 0.43
		2	2.93 ± 0.25	6.14 ± 0.48	11.09 ± 0.81	
		3	2.59 ± 0.22	5.72 ± 0.42	11.41 ± 0.53	
CBG + CBGA	10.49 ± 0.75	1	3.11 ± 0.11	5.87 ± 0.44	10.01 ± 0.69	9.68 ± 0.30
		2	3.06 ± 0.95	6.01 ± 0.58	9.62 ± 0.19	
		3	2.47 ± 0.61	5.98 ± 0.65	9.41 ± 0.55	
	9.17 ± 0.76	1	2.96 ± 0.49	6.02 ± 0.61	9.51 ± 0.38	9.54 ± 0.18
		2	2.73 ± 0.65	5.47 ± 0.38	9.75 ± 0.42	
		3	3.22 ± 0.47	5.13 ± 0.63	9.39 ± 0.54	
	9.87 ± 0.47	1	2.94 ± 0.15	5.84 ± 0.34	9.52 ± 0.52	9.55 ± 0.04
		2	2.40 ± 0.45	5.95 ± 0.57	9.60 ± 0.51	
		3	2.99 ± 0.59	5.48 ± 0.60	9.55 ± 0.43	

CBD: cannabidiol, CBDA: cannabidiolic acid, CBG: cannabigerol and CBGA: cannabigerolic acid
